# Refining the psychometric properties of the Trinity Student Occupational Performance Profile – A self-report measure of occupational performance difficulties within the student role

**DOI:** 10.1177/03080226221107762

**Published:** 2022-06-20

**Authors:** Kim Lombard, Clodagh Nolan, Elizabeth Heron

**Affiliations:** 1 Discipline of Occupational Therapy, 8809 Trinity College Dublin, Dublin, Ireland; 2 Department of Psychiatry, 155276 Trinity College Dublin, Dublin, Ireland

**Keywords:** self-report assessment, Rasch analysis, occupational performance, students with disabilities, third-level education, Person-Environment-Occupation model

## Abstract

**Introduction:**

Navigating the transition to university can pose occupational performance difficulties for students with mental health disabilities including those on the autism spectrum or with attention deficit hyperactivity disorder. This study aimed to refine the Trinity Student Occupational Performance Profile (TSOPP) – a self-report measure of occupational performance difficulties within the student role for students with mental health disabilities which is based on the Person-Environment-Occupation model.

**Method:**

Data from 667 files were gathered from two Irish universities. Rasch analyses were conducted on the measure’s item-sets (i.e. Person *N* = 30; Environment *N* = 20; Occupation *N* = 24) and on an item-set which combined all 74 items. All item-sets were assessed for fit, rating scale functioning, dimensionality, reliability and separation indices.

**Results:**

The TSOPP demonstrated stronger psychometric properties as a combined item-set which measures the ultimate construct of occupational performance difficulties within the student role. The 6-point scale was collapsed into a 4-point scale and 20 redundant items were removed. The item difficulty hierarchy provided empirical evidence for occupational performance difficulties in the student role.

**Conclusion:**

The TSOPP is a valid and reliable self-report measure of occupational performance difficulties within the student role for students with mental health disabilities in higher education.

## Introduction

It is estimated that globally, one third of newly entered college students experience difficulties with their mental health (Auerbach et al., 2018). Transitioning to higher education requires students to make academic, social and personal adjustments as they navigate the autonomous institutional environment of university (Baker and Siryk, 1984), all of which impact on a student’s occupational performance (Keptner and Rogers, 2018) and satisfaction with occupational performance (Keptner, 2018). These occupational performance difficulties can include difficulties with social-related occupations, time management, academic-related occupations, sleep, managing stress and managing money (Keptner and Rogers, 2018). Furthermore, the transition to higher education may be accompanied by additional occupational performance difficulties for students with disabilities such as mental health difficulties (Storrie et al., 2010) including autism spectrum disorder (ASD; Nuske et al., 2019) and attention deficit hyperactivity disorder (ADHD; Jansen et al., 2017). Supporting the transition of students with mental health disabilities is an emerging area of practice for occupational therapists (Spencer et al., 2018). Hence, it is imperative that assessment practices reliably and efficiently identify students’ occupational performance difficulties within their student role so that appropriate reasonable accommodations and interventions can be implemented in a time-sensitive manner. However, widely used measures of occupational performance do not specifically investigate the nuanced occupational performance difficulties experienced within the student role. For example, the *Occupational Self-Assessment* (Kielhofner and Forsyth, 2001) refers to the student role alongside other roles such as the worker, volunteer or family member role but does not examine this role in detail. Moreover, Keptner and Rogers (2018) used the *Canadian Occupational Performance Measure* (Law et al., 2005) to investigate the occupational performance concerns of the general student population but needed to adapt the measure to include questions which were more relevant to university students. Keptner (2018) further highlighted the need for a tool which accurately identifies a student’s occupational performance concerns and self-perceptions of performance.

A tool that fulfils this need is the Trinity Student Occupational Performance Profile (TSOPP), previously known as the Trinity Student Profile (TSP; Nolan, 2011), which is a self-report measure of occupational performance difficulties for students with mental health disabilities in higher education. The measure was developed in response to the increasing academic, social and personal difficulties experienced by students with mental health disabilities in managing their student role in higher education (Nolan, 2011). The tool has not been published in a peer-review journal to-date but has been utilised in several occupational therapy services in higher education in Ireland (Nolan, 2011). The TSOPP is underpinned by the Person-Environment-Occupation model (PEO; Law et al., 1996) which provides an appropriate framework for students to identify their occupational performance difficulties and understand the transactive relationship between person factors (e.g. cognitive and affective factors; physical factors will not be discussed in this article), their university environment (e.g. physical, social, cultural and institutional environments) and the occupational demands of being a student (e.g. academic, social and personal occupations). The TSOPP consists of the following five parts, of which the first three are self-reported by the student and parts four and five are completed in collaboration with the therapist:

• Part One ‘Student Details’ captures demographic details including contact details, course, year of study, next of kin and psychiatrist/GP details if applicable.
• Part Two ‘Experiences and Expectations’ asks open-ended questions regarding a students’ strengths, hobbies/interests, college and work experience followed by what their expectations are academically, socially and personally for the year. Furthermore, the PEO-model (Law et al., 1996) diagram is displayed to inform students of the conceptual model underlying the approach to the self-report measure.
• Part Three ‘Identifying Needs’ enables students to self-report their occupational performance difficulties within the student role. There are 74 items split over Person (*N* = 30), Environment (*N* = 20) and Occupation (*N* = 24) item-sets and students rate how difficult an item is to manage on a 6-point Likert-style ‘Difficulty’ scale.
• Part Four ‘Course Demands (Module Matrix)’ allows students and therapists to identify the demands which they are expected to meet within each module of the student’s course (e.g. assignments, exams and placement/internship).
• Part Five ‘Goal-setting’ enables students and therapists to identify priorities for therapy and collaboratively set goals.

As the scope of this study was on refining the psychometric properties of the TSOPP, this article will only discuss the 6-point Likert-style ‘Difficulty’ scale in Part Three ‘Identifying Needs’ from here on.

In establishing preliminary psychometric properties of Part Three’s 6-point ‘Difficulty’ scale, Nolan (2011) used a mixed-methods approach to develop and pilot the original paper-based measure. During Nolan’s (2011) pilot, data from 140 ‘Difficulty’ scales were analysed using Classical Test Theory. Following a clinical audit to improve fidelity and adherence of the service to standards of practice, the paper-based measure was converted to an electronic Excel-based format without modifying item content. The new version was known as the *e*TSP and was fully rolled out in 2015 (Creaner and Nolan, 2016).

Although this version of the self-report TSOPP has been used in practice, the psychometric properties of Part Three ‘Identifying Needs’ require further rigorous validation and refinement. This is especially important as the construct it intends to measure (i.e. self-reported occupational performance difficulties within the student role) is latent, meaning it is abstract and cannot be easily observed (Bond and Fox, 2015). Furthermore, Paulhus and Vazire (2007) raise concerns regarding the credibility and sources of bias of self-report measures such as the TSOPP, arguing that clients may choose socially desirable answers or may lack self-awareness. Smith et al. (2003) also highlight how some clients are prone to choose extreme scores on a Likert-style scale while others may only choose middle categories. Self-report measures which have poorly defined category labels or have too many categories to discriminate between may lead to clients using the scale idiosyncratically which can affect the measure’s validity (Smith et al., 2003). For the TSOPP to be robust in measuring a latent construct such as occupational performance difficulties, the items should be well-defined and act as a ruler or hierarchy, representing ‘less’ to ‘more’ of the construct (Bond and Fox, 2015). Consequently, this ruler or hierarchy would enable the identification of where a student sits on this hierarchy (i.e. a student’s level of occupational performance difficulties) and would subsequently assist in developing graded intervention plans. Therefore, the aim of this study was to improve the reliability and validity of the TSOPP by refining the psychometric properties of the self-report ‘Difficulty’ scale in Part Three ‘Identifying Needs’ using Rasch analysis (Bond and Fox, 2015; Lombard et al., 2021).

## Method

### Sample

This study retrospectively collected 667 irrevocably anonymised Part Threes created between 2007 and 2017 from the disability services in two large Irish universities using purposive and convenience sampling. Ethical approval was obtained from both universities, and written consent was deemed unnecessary by the ethics boards as the data was irrevocably anonymised. Sample demographics are outlined in Table 1. The occupational therapy service was established in University One (85.3%, *N* = 569) in 2004, whereas the service was only established in 2012 on a part-time basis in University Two (14.7%, *N* = 98).

**Table 1.** Sample demographics (*N* = 667).

Sample demographic	*N* = 667
Gender	
Male	323 (48.4%)
Female	341 (51.1%)
Missing	3 (0.5%)
University	
One	569 (85.3%)
Two	98 (14.7%)
Format	
Paper (trinity student profile)	444 (66.6%)
Excel (*e*trinity student profile)	223 (33.4%)
Age (years)	
Range	17-46
Mean (SD)	22.85 (5.7)
Mode	19
Level of degree	
Undergraduate	594 (89.1%)
Postgraduate	68 (10.2%)
Missing	5 (0.7%)
Disability	
Depression	170 (25.5%)
Asperger’s syndrome/autism spectrum disorder	114 (17.1%)
Attention Deficit Disorder (ADD)/Attention Deficit Hyperactivity Disorder (ADHD)	76 (11.4%)
Anxiety	67 (10.0%)
Mental health other	101 (15.1%)
Dyspraxia/Developmental Coordination Disorder (DCD)/specific learning difficulty	61 (9.2%)
Other (physical, sensory, significant ongoing illness, neurological, speech and language)	75 (11.2%)
Missing	3 (0.5%)
Repeating (e.g. modules, entire year)	
No	505 (75.7%)
Yes	115 (17.2%)
Missing	47 (7.1%)
Faculty	
Arts, humanities, Social science and law, business	401 (60.2%)
Science, engineering, mathematics and architecture	163 (24.4%)
Health and agricultural sciences	95 (14.2%)
Missing	8 (1.2%)
Year of study	
1^st^ year/access course	312 (46.8%)
2^nd^ year	116 (17.4%)
3^rd^ year	99 (14.8%)
4^th^ year/5^th^ year medicine	64 (9.6%)
Postgrad masters/PhD/diploma	68 (10.2%)
Missing	8 (1.2%)

### Instrument

Part Three ‘Identifying Needs’ consists of 74 items split over three item-sets based on the Person (*N* = 30), Environment (*N* = 20) and Occupation (*N* = 24) model (Law et al., 1996). The original author of the self-report measure (Nolan, 2011) intended for students to indicate how difficult each item is to manage by rating them on a 6-point Likert-style ‘Difficulty’ scale (i.e. *0* = *no difficulty; 5* = *extreme difficulty*). However, the adjectival descriptors of the middle categories are not displayed on the measure (i.e. *1 = some difficulty, 2 = small difficulty, 3 = medium difficulty and 4 = moderate difficulty*). It is acknowledged that the ultimate construct of the underlying PEO-model is occupational performance (Law et al., 1996). Hence, in addition to the separate Person, Environment and Occupation item-sets, this study evaluated a combined item-set in which all 74 ‘Difficulty’ items were analysed together to determine if the TSOPP demonstrates stronger psychometric properties as one combined scale of occupational performance difficulties or as separate Person, Environment and Occupation scales.

### Data analysis

The Rasch model is a probabilistic model which assumes that the responses a student gives to each item on the TSOPP are a function of the item’s ‘difficulty’ and the person’s ‘ability’ (Wright and Masters, 1982). Rasch analysis is a person-centred measurement model (Velozo, 2021) which enables the relative difficulty of each item (i.e. item difficulty measure) to be reflected on a hierarchy from ‘less’ to ‘more’, similar to a ruler (Bond and Fox, 2015). Simultaneously, Rasch analysis can determine where a student is situated (i.e. person measure) on the ‘occupational performance difficulty’ hierarchy (Velozo, 2021), even if there is missing data (Smith and Wind, 2018). This is advantageous over traditional total raw scores which may suggest that a student has lower levels of occupational performance difficulty when in fact they have just left several items unanswered. The data was inputted on site of both disability services and analysed using WINSTEPS version 4.7.0.0 (Linacre, 2020a). As all 74 items share a common 6-point scale, it was determined that the Rating Scale Model was the most appropriate model to use for the analysis (Andrich, 1978; Wright and Masters, 1982).

#### Item and person fit

The Rasch model has expectations for how students respond to TSOPP items. For example, it expects students with high levels of occupational performance difficulties to choose higher rating scale categories for most items, whereas students who are managing well are expected to choose lower rating scale categories. Fit statistics (i.e. mean square fit statistics [*MnSq*] and standardised mean square fit statistics [*Zstd*]) indicate how well item-related data or person-related data fits these expectations. Data which has a *MnSq* > 1.4 and a *Zstd* > 2.0 are considered to be misfitting (Bond and Fox, 2015). An item might misfit if it is measuring a different construct to the rest of the items (i.e. multidimensionality). A person might misfit if they have an erratic response pattern, such as choosing ‘*0 = no difficulty’* for very difficult items and ‘*5 = extreme difficulty’* for very easy items. It is expected that 5% of data misfits by chance (Smith, 2002) and can be dealt with by removal if warranted. This study also sought to determine the impact that misfitting persons had on item difficulty measures by assessing displacement (Linacre, 2020b). This process involves calibrating item difficulty measures while excluding misfitting persons, re-instating the misfitting persons into the analysis, re-calibrating the item difficulty measures and then determining by how much the item difficulty measures were moved or displaced by the misfitting persons. Any displacement between ±0.5 logits is considered inconsequential, while displacements >0.5 logits or <−0.5 logits are considered significant (Linacre, 2020b), with the logit being the unit of measurement used in Rasch analysis (Bond and Fox, 2015).

#### Rating scale functioning

Within the context of the TSOPP, the Rating Scale Model (Andrich, 1978; Wright and Masters, 1982) assumes that students with increasingly higher levels of occupational performance difficulties will choose increasingly higher rating scale categories to reflect this. This is known as ordered categories. Disordering of categories can occur if there are too many categories for people to adequately differentiate between (Weng, 2004), or if some categories are not used frequently in practice (Linacre, 2001). Linacre (2004) outlines guidelines for assessing the functioning of a rating scale: assessing if each category satisfies the following (a) has >10 observations, (b) the average person measure increases as the categories increase, (c) the *MnSq*<2.0 and (d) that the threshold between each category (i.e. the point at which a student has equal probability of choosing adjacent categories) increases as the categories increase. Categories may be collapsed together to remedy category disordering (Bond and Fox, 2015).

#### Dimensionality

Unidimensionality indicates that items work together to measure one construct (Bond and Fox, 2015), such as occupational performance difficulty for the TSOPP. Two methods of assessing dimensionality include conducting a principal component analysis of the residuals and assessing local independence. The principal component analysis of residuals is used to determine if there were any unexpected patterns which would indicate multidimensionality, this would be indicated if there was >5% unexplained variance with an eigenvalue >2.0 (Bond and Fox, 2015). For TSOPP items to be locally independent (Yen, 1993), the response a student gives to one item should not influence the response they give to another item. Items may violate local independence if they are similarly worded or measure similar concepts and these violations can be remedied by removing redundant items (Bond and Fox, 2015). Item pairs with an inter-item correlation >0.4 were investigated (Linacre, 2020c).

#### Reliability and separation

In Classical Test Theory, the reliability of a measure is represented by Cronbach’s alpha, as in Nolan’s (2011) pilot study of the TSOPP. In Rasch analysis a similar indicator, the person reliability index, which ranges from 0 to 1, is used (Bond and Fox, 2015). Similarly, an item reliability index, which also ranges from 0 to 1, indicates the extent to which the item difficulty hierarchy would be perceived consistently across different samples (Bond and Fox, 2015). For separation, the person separation index indicates how well a measure can distinguish between differing levels of the construct among the sample, while the item separation index indicates how many levels of difficulty exist among the items (Wolfe and Smith, 2007). For this study, a person reliability index >0.80 with a person separation index >2 and an item reliability index >0.90 with an item separation index >3 is considered acceptable (Bond and Fox, 2015). Cronbach’s alpha between 0.7 and 0.95 was considered acceptable (Terwee et al., 2007).

#### Item difficulty hierarchy and targeting

In Rasch analysis, an item difficulty hierarchy can be generated which empirically orders items from ‘less’ to ‘more’ of a construct (Wolfe and Smith, 2007). For the TSOPP, the item difficulty hierarchy is ideal for providing insight into the relative difficulty of the items which reflect occupational performance difficulties associated with the occupational role of being a student (Lombard et al., 2021). The item difficulty hierarchy can be displayed using a person-item map. This map allows for the identification of ceiling or floor effects and to determine if the range of difficulty of the items sufficiently captures the range of occupational performance difficulties experienced by the students in the sample, also known as targeting (Bond and Fox, 2015).

## Results

Using Rasch analysis, the TSOPP underwent a six-step iterative refinement process outlined below.

### Step One

Initial Rasch analyses of the respective Person, Environment and Occupation item-sets as well as the combined TSOPP item-set were conducted (Table 2). The 6-point rating scale did not function optimally across any of the item-sets, with evidence of disordering between categories *‘1 = some difficulty’* and ‘*2 = small difficulty’*. Furthermore, the Environment item-set demonstrated an inadequate person reliability index (0.76) and person separation index (1.80). Greater than 5% of items misfit in the separate Person (2/30, 6.7%), Environment (1/20, 5%) and Occupation (2/24, 8.3%) item-sets, indicating multidimensionality. However, the combined item-set demonstrates no evidence of item misfit, indicating that all 74 items are working well together to measure the construct of occupational performance difficulty in the student role. Person misfit appears to be an issue for each item-set.

**Table 2.** Initial Rasch analysis of ‘Difficulty’ scale.

	Item-set	Person (*N* = 30)	Environment (*N* = 20)	Occupation (*N* = 24)	Combined (*N* = 74)	Combined w/4-point scale (*N* = 74)	Refined 4-point scale (*N* = 54)
	Sample size	665	662	661	667	667	667
	Rating scale categories	6	6	6	6	4	4
Rating scale (Linacre 2004)	>10 observations/category	Yes	Yes	Yes	Yes	Yes	Yes
	Average measure increases	Yes	Yes	Yes	Yes	Yes	Yes
	*MnSq* <2.0	Yes	Yes	Yes	Yes	Yes	Yes
	Andrich threshold increases	**No**	**No**	**No**	**No**	Yes	Yes
Reliability and separation	Person reliability	0.88	**0.76**	0.86	0.94	0.93	0.91
	Person separation	2.65	**1.80**	2.49	3.91	3.79	3.15
	SE of person mean	0.02	0.02	0.02	0.01	0.02	0.02
	Cronbach’s alpha	0.90 (SEM 7.59)	0.84 (SEM 6.29)	0.91 (SEM 6.97)	0.95 (SEM 12.40)	0.93 (SEM 7.14)	0.91 (SEM 6.06)
	Item reliability	0.99	0.99	0.99	0.99	0.99	0.99
	Item separation	12.4	9.80	10.55	13.46	13.25	13.90
	SE of item mean	0.07	0.07	0.07	0.05	0.08	0.10
Item fit	Misfitting items (*MnSq*>1.4, *Zstd*>2.0)	**2/30 (6.7%)**	**1/20 (5%)**	**2/24 (8.3%)**	0	0	0
Person fit	Misfitting persons (*MnSq*>1.4, *Zstd*>2.0)	**75 (11.3%)**	**60 (9.1%)**	**69 (10.4%)**	**98 (14.7%)**	**104 (15.6%)**	102 (15.3%) (accepted)
Dimensionality and local independence	Inter-item correlation >0.40	**9 item pairs**	**5 item pairs**	**7 item pairs**	**37 item pairs**	**34 item pairs**	2 item pairs (accepted)
	PCA: Unexplained variance >5% and eigenvalue >2.0	**3**	**1**	**3**	0	**1**	0

**Key:** Bold = issues with psychometric properties; *MnSq* = mean square fit statistic; *Zstd* = standardised mean square fit statistic; SE = standard error; SEM = standard error of measurement; PCA = principal component analysis

The principal component analysis of the residuals of the separate item-sets indicates multidimensionality, whereas the combined TSOPP item-set demonstrates unidimensionality with no contrasts having an eigenvalue >2.0 with unexplained variance >5%. For local independence, by combining the TSOPP items together, it is possible to identify the local independence issues which arise between items across the Person, Environment and Occupation item-sets due to the transactive nature of the PEO, which would otherwise not have been identified. For example, ‘*Handing up work on time [HANDWORK]’* (Person item-set) violated local independence with ‘*Dealing with time pressures and deadlines [PRESSDEA]’* (Occupation item-set). Considering the item content, this violation of local independence is not surprising. This supports the argument that the TSOPP ‘Difficulty’ scale demonstrates stronger psychometric properties as one combined scale of occupational performance of the student role in comparison to separate Person, Environment and Occupation scales because of the transactive relationship between the person, environment and occupation within the PEO-model (Law et al., 1996). Hence, subsequent analyses focus on remedying the issues associated with this combined scale.

### Step Two

**Figure 1.**
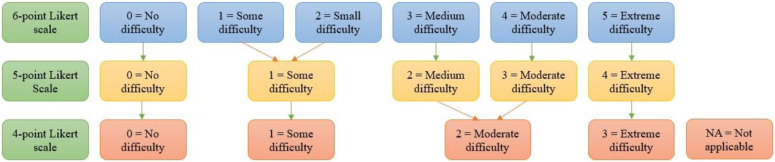
Refinement of 6-point ‘Difficulty’ Likert scale to a 4-point Likert scale.

The 6-point Likert-style ‘Difficulty’ rating scale demonstrated evidence of category disordering (see Supplementary Table 1). Figure 1 outlines how the 6-point Likert scale was collapsed into a 4-point Likert scale which meets Linacre’s (2004) rating scale functioning requirements. A ‘not applicable’ option has been added to give students the ability to indicate items which are not applicable to their specific course (e.g. placement/lab items for students in Arts & Humanities; exam-related items for postgraduate students who do not have exams).

### Step Three

After the rating scale was remedied, 104 (15.6%) persons misfit (Table 2). A descriptive analysis did not highlight major differences between these misfitting persons and the remainder of the sample. When determining the impact of misfitting persons on the item difficulty measures, it was found that the item difficulty measure displacements ranged from −0.28 logits to 0.16 logits which is considered inconsequential (Linacre, 2020b). This indicates that misfitting persons did not substantially change the item difficulty measures; hence, all misfitting persons were retained for subsequent analyses.

### Step Four

The first contrast in the principal component analysis of the residuals of the 4-point 74-item ‘Difficulty’ scale had an eigenvalue of 5.85 with unexplained variance of 5.0% (Table 2), indicating potential multidimensionality (Bond and Fox, 2015). Thirty-four item pairs violated local independence with inter-item correlations ranging from 0.4 to 0.71 which contributed to this multidimensionality. To remedy these violations, redundant items were removed stepwise. A total of 20 items (see Supplementary Table 2) were removed resulting in a 54-item scale. Items from two pairs which violated local independence were retained as they were deemed to be clinically relevant (i.e. ‘*Managing alcohol intake [MANALCOH]*’ and ‘*Managing/avoiding other substances [MANSUBST]*’and ‘*Participating in discussion [PARTDISC]*’ and ‘*Doing presentations [PRESENTA]*’). Remedying the local independence violations subsequently remedied the potential multidimensionality, with the principal component analysis of the residuals of the 54-item TSOPP being within acceptable ranges. All 54 items fit the model’s expectations (see Supplementary Table 3).

### Step Five

The 4-point 54-item TSOPP ‘Difficulty’ scale had a person reliability index of 0.91 and person separation index of 3.15, while the item reliability index was 0.99 and item separation index was 13.90, all indicating excellent reliability and separation (Bond and Fox, 2015). Cronbach’s alpha was 0.91 which is considered strong (Terwee et al., 2007).

### Step Six

**Figure 2.**
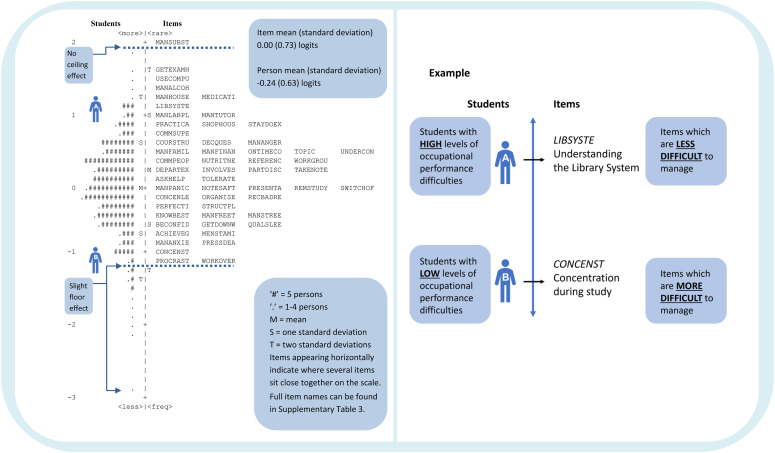
Person-item map of the 54-item 4-point Trinity Student Occupational Performance Profile ‘Difficulty’ scale.

The item difficulty hierarchy is illustrated using a person-item map (Figure 2). This map demonstrates the relative level of occupational performance difficulties of students in the sample on the left-hand side and the relative difficulty of the items on the right-hand side. The example provided demonstrates that student A is experiencing high levels of occupational performance difficulties in comparison to student B. Student A is finding the item ‘*Understanding the Library System*’ difficult to manage and hence it is likely that they are experiencing difficulty with all items that fall below this item. However, student B is managing most of the items but is having difficulty managing some of the most difficult items such as ‘*Concentration during study [CONCENST]*’. The item difficulty hierarchy for the TSOPP confirms that items relating to affective factors (e.g. ‘*Managing anxiety [MANANXIE]*’, ‘*Maintaining mental stamina and endurance [MENSTAMI]’*) and executive functioning (e.g. ‘*Procrastination [PROCRAST]’* and ‘*Dealing with work overload [WORKOVER]*’) are some of the most difficult to manage within the student role.

This person-item map demonstrates that the TSOPP is well targeted to the sample as there is a sufficient spread of items along the scale (i.e. right-hand side) to adequately capture the range of occupational performance difficulties experienced by students in the sample (i.e. left-hand side). There is no evidence of a ceiling effect in the items as the item *‘Managing/avoiding other substances [MANSUBST]’* is calibrated higher than the person with the highest level of occupational performance difficulties. Although there is a slight floor effect, it is likely that students falling at this end of the scale are experiencing low levels of occupational performance difficulties and would not require occupational therapy intervention. Hence, this slight floor effect is not a concern from a clinical perspective.

## Discussion and implications

Using Rasch analysis, this study investigated and refined the psychometric properties of Part Three ‘Identifying Needs’ of the TSOPP. This investigation indicated that there were issues residing with the separate Person, Environment and Occupation item-sets which were mitigated in the combined item-set. Item fit and dimensionality were an issue for the separate item-sets which suggests potential multidimensionality (Bond and Fox, 2015). Considering the components of the underlying PEO-model (Law et al., 1996), this multidimensionality is not unexpected. The ‘Person’ consists of cognitive and affective components; the ‘Environment’ encompasses the physical, social, cultural and institutional components and the ‘Occupation’ consists of tasks, activities and occupations (Canadian Association of Occupational Therapy, 1997). One method of remedying item misfit is item removal (Wright and Masters, 1982). However, the items which misfit in the Person (‘*Understanding the Library System [LIBSYSTE]’; ‘Handing up work on time [HANDWORK]’*), Environment (‘*Tolerating external distractions [TOLERATE]’*) and Occupation (‘*Staying and doing the exams [STAYDOEX]’; ‘Doing practical work [PRACTICA]’*) item-sets are all clinically relevant and if these items were removed with the only aim of remedying item misfit, valuable clinical information would be lost. Conversely, when the items were combined, there was evidence of item fit and unidimensionality, meaning the items were working well together to measure the ultimate construct of the PEO-model, namely, difficulties with occupational performance (Law et al., 1996). Furthermore, there were local independence violations in each item-set with evidence of violations between items from across the original Person, Environment and Occupation item-sets in the combined item-set. This makes sense as there is an overlapping transactive relationship between the person, environment and occupation concepts within the PEO-model (Law et al., 1996). However, these violations would not have been highlighted if the TSOPP items were not combined. As for reliability and separation, the combined item-set demonstrated stronger person and item reliability and separation as well as Cronbach’s alpha than the separate item-sets (Table 2). In Nolan’s (2011) original pilot study of the measure, Cronbach’s alpha ranged from 0.518 to 0.887 with the Environment item-set demonstrating the weakest reliability. In this study, the Environment item-set demonstrated inadequate person reliability and separation. However, by combining all items into a combined scale of occupational performance difficulty, these issues were mitigated. Consequently, it is evident that the TSOPP demonstrates stronger psychometric properties as one combined scale measuring occupational performance difficulties within the student role.

Nevertheless, the combined item-set posed some measurement challenges which were resolved in this study. In relation to the rating scale functioning, the 6-point scale had evidence of disordered categories. Although Preston and Colman (2000) demonstrated that increasing the rating scale categories may increase reliability, Weng (2004) argues that having too many categories makes it more complex as it is difficult to discriminate between adjacent categories. This was the case for the TSOPP’s ‘Difficulty’ scale as six categories appeared to be too many for students to adequately discriminate between and the scale was collapsed to a 4-point scale. In practice, this shorter scale should make it easier for students to accurately identify their occupational performance difficulties. Nevertheless, future research should validate this refined rating scale (Smith et al., 2003) using a new sample of students with mental health disabilities in higher education.

In addition to the reduction in the rating scale categories, the number of items in the TSOPP was reduced from 74 to 54. Local independence violations may occur due to potential multidimensionality or items which are measuring similar concepts or have similar wording (Yen, 1993). As local independence between items is an assumption of the Rasch model (Bond and Fox, 2015), these violations needed to be remedied even though the TSOPP demonstrated strong person and item reliability and Cronbach’s alpha as a 74-item scale. Prior to removing the redundant items, Cronbach’s alpha was 0.93 while after it was 0.91. However, Terwee et al. (2007) describe how Cronbach’s alpha is impacted by the number of items in the scale and that extremely high indicators can be the result of high correlations and redundancy between items. Hence, Cronbach’s alpha of 0.91 is still considered excellent (Terwee et al., 2007). As for person and item reliability and separation, although there were slight reductions in some of these indicators, all were within acceptable ranges (Bond and Fox, 2015). Moreover, all 54 items demonstrated appropriate fit statistics, meaning they are working well together to measure the construct of occupational performance difficulties within the student role (Wright and Masters, 1982). In practice, this shortening of the scale will reduce the length of time for students to complete the TSOPP, minimising the burden of response (Prieto et al., 2003). As for person misfit, although it was demonstrated that misfitting persons did not substantially impact the item difficulty measures or hierarchy, a larger proportion (*N* = 104, 15.6%) of persons misfit than the 5% that was expected by chance (Smith, 2002). Hence, future research should further investigate person fit with another sample of students with mental health disabilities in higher education, both investigating person fit statistics and clarifying response patterns with students qualitatively to better understand the cause of the misfit.

This study illustrated the item difficulty hierarchy of occupational performance difficulties within the student role, a novel finding which is beneficial both theoretically and clinically. Although literature exists regarding the occupational performance difficulties experienced by students with mental health disabilities in university (Jansen et al., 2017; Nuske et al., 2019; Storrie et al., 2010), this study has been able to provide novel evidence regarding the relative difficulty of these challenges. For example, items relating to affective factors and executive functioning were the most difficult to manage, which reflects the continuing brain development occurring between adolescence and adulthood as young people try to attain the necessary independence skills (Spear, 2000). This hierarchy not only adds to theory and understanding regarding the relative difficulty of occupational performance within the student role but also enables therapists to better grade intervention plans with students. For example, therapists and students may discuss this hierarchy and a student’s level of reported occupational performance difficulties and start intervention with a just-right challenge (Christie, 1999; Velozo, 2021).

### Limitations

The results must be considered within the study’s methodological limitations. Although using existing anonymised data allowed a large sample to be gathered easily to conduct Rasch analysis, it was not possible to clarify response patterns with students to better understand erratic response patterns and hence identify potential sources of person misfit. Furthermore, as the data was gathered from only two Irish universities, this limits the generalisability of the results to other universities in Ireland or internationally. Moreover, the sample consisted of TSOPPs completed by students who were formally registered with the university disability services and hence may not fully reflect the occupational performance difficulties experienced by students with mental health disabilities who are not registered with these services.

### Recommendations for future research

As mentioned previously, further research should look to validate this study’s findings using an independent sample of students with mental health disabilities in higher education. Moreover, future research may determine the validity of using the TSOPP with the general student population, especially in determining if any differences with occupational performance difficulties exist between the general student population and those who have identified with a mental health disability. Rasch analysis also enables the creation of keyforms, paper-and-pencil forms which allow therapists to estimate a student’s level of occupational performance difficulty without the need for complex Rasch analysis software (Linacre, 2020a). Keyforms ensure that the numbers generated by a measure are relevant and useful in practice by supporting the identification of just-right challenges for intervention as well as enhancing a student’s self-awareness about their occupational performance difficulties (Velozo, 2021). Furthermore, to provide evidence for the generalisability (Wolfe and Smith, 2007) of the TSOPP, future research should investigate if the TSOPP can be used as an outcome measure (e.g. is it sensitive to detecting change over time) or if the item difficulty hierarchy is interpreted the same across different groups (e.g. university, disability, culture, gender, year and faculty). Finally, conducting qualitative research to gather the perceptions of occupational therapists in using the refined TSOPP in practice can provide evidence for its usability.

## Conclusion

The refined TSOPP presented here is a valid and reliable self-report measure which adequately captures the occupational performance difficulties of students with mental health disabilities within their student role in university. The refined 4-point 54-item ‘Difficulty’ scale enables students to self-identify their level of occupational performance difficulties. The item difficulty hierarchy is beneficial both theoretically and clinically as it furthers knowledge regarding the relative difficulty of the tasks, activities and occupations associated with managing the student role in higher education. The refined TSOPP provides occupational therapists seeking to support students with mental health disabilities in university with a credible measure to guide their practice. Future validation research will add to the evidence base of the TSOPP, while developing a user manual will assist therapists unfamiliar with the measure in how to appropriately administer it in practice.

## Key findings

• The TSOPP demonstrates strong psychometric properties as one scale measuring occupational performance difficulties within the student role.
• The Rasch analysis refinement process resulted in a 54-item 4-point TSOPP ‘Difficulty’ scale.

## What the study has added

Rasch analysis enabled the refinement of the TSOPP’s psychometric properties and provided novel insight into the item difficulty hierarchy of occupational performance difficulties within the student role in higher education.

## Supplemental Material

sj-pdf-1-bjo-10.1177_03080226221107762 – Supplemental Material for Refining the psychometric properties of the Trinity Student Occupational Performance Profile – A self-report measure of occupational performance difficulties within the student role Supplemental Material, sj-pdf-1-bjo-10.1177_03080226221107762 for Refining the psychometric properties of the Trinity Student Occupational Performance Profile – A self-report measure of occupational performance difficulties within the student role by Kim Lombard, Clodagh Nolan and Elizabeth Heron in British Journal of Occupational Therapy
