# Supplementary material for: Refining the psychometric properties of the Trinity Student Occupational Performance Profile – A self-report measure of occupational performance difficulties within the student role
Source: Br J Occup Ther. 2022 Jun 20;86(4):265–74. doi: 10.1177/03080226221107762 (PMC12033774; doi:10.1177/03080226221107762)
Supplement: sj-pdf-1-bjo-10.1177_03080226221107762 – Supplemental Material for Refining the psychometric properties of the Trinity Student Occupational Performance Profile – A self-report measure of occupational performance difficulties within the student role [file sj-pdf-1-bjo-10.1177_03080226221107762.pdf]

Supplementary Information for:

Refining the Psychometric Properties of the Trinity Student Occupational Performance Profile (TSOPP) – A self-report measure of occupational performance difficulties within the student role.

Supplementary Table 1: Summary of Rating Scale Functioning of the 'Difficulty' Scale.

	6-point rating scale						5-point rating scale					4-point rating scale			
	0	1	2	3	4	5	0	1	2	3	4	0	1	2	3
Frequency	11386	5447	6327	7878	7520	7764	11386	11774	7878	7520	7764	11386	11774	15398	7764
N (%)	(25%)	(12%)	(14%)	(17%)	(16%)	(17%)	(25%)	(25%)	(17%)	(16%)	(17%)	(25%)	(25%)	(33%)	(17%)
Average Measure	-0.58	-0.31	-0.15	0.02	0.18	0.37	-0.78	-0.35	-0.04	0.18	0.44	-0.99	-0.36	0.15	0.63
Outfit MnSq	0.97	1.06	0.91	0.99	1.09	1.08	0.96	0.98	0.92	1.10	1.13	0.98	0.95	1.01	1.09
Andrich Threshold	None	**0.28**	**-0.40**	-0.28	0.16	0.25	None	-0.59	**0.18**	**0.11**	0.30	None	-0.71	-0.38	1.09

Key: Bold = decrease in Andrich Threshold indicating disordered categories; *MnSq* = mean square fit statistic

Supplementary Table 2: Item pairs violating local independence and subsequent resolutions.

Iteration	Items violating local independence	Inter-item correlation	Resolution
1	40 – Communicating with other students [E]	0.71	Removed item 40
	38 – Communicating with people [E]		
2	5 – Retrieving information/books [P]	0.71	Removed item 5
	4 – Understanding the Library System [P]		
3	51 – Participating in discussion [O]	0.62	Removed item 52
	52 – Asking questions [O]		
4	41 – Making friends within college [E]	0.62	Removed item 41
	42 – Making friends outside college [E]		
5	11 – Getting started with studying [P]	0.61	Removed item 11
	12 – Procrastination [P]		
6	64 – Managing work load [O]	0.60 (items 64 & 63)	Removed items 64 & 65
	65 – Completing reports [O]	0.51 (items 64 & 65)	
	63 – Doing practical work [O]	0.46 (items 63 & 65)	
7	17 – Recall material [P]	0.59	Removed item 17
	14 – Remembering what I have studied [P]		
8	55 – Talking to lecturers and tutors [O]	0.54	Removed item 55
	56 – Asking for help [O]		
9	21 – Managing anxiety [P]	0.53	Removed item 22
	22 – Managing negative thoughts [P]		
10	21 – Managing anxiety [P]	0.53	Removed item 23
	23 – Managing stressful situations [P]		
11	15 – Managing the stress before an exam [P]	0.52	Removed item 19
	19 – Managing fear that I may fail exams [P]		
12	68 – Finishing the work [O]	0.49	Removed item 68
	67 – Continuing writing, avoiding “writer’s block” [O]		
13	9 – Handing up work on time [P]	0.48	Removed item 9
	57 – Dealing with time pressures and deadlines [O]		
14	38 – Communicating with people [E]	0.46	Removed item 42
	42 – Making friends outside of college [E]		

15	61 – Balancing college work and life [O]<br>62 – Managing my free time [O]	0.46	Removed item 61
16	26 – Managing conflict [P]<br>27 – Managing anger [P]	0.44	Removed item 26
17	35 – Managing student support services [E]<br>36 – Managing Tutor system [E]	0.43	Removed item 35
18	58 – Goal-setting [O]<br>59 – Achieving goal [O]	0.42	Removed item 58
19	49 – Managing alcohol intake [E]<br>50 – Managing/avoiding other substances [E]	0.42	Both items retained Due to clinical relevance
20	66 – Getting down to writing [O]<br>67 – Continuing writing, avoiding “writer’s block” [O]	0.41	Removed item 67
21	51 – Participating in discussion [O]<br>54 – Doing presentations [O]	0.41	Both items retained

Key: [ ] indicates which item-set the item originated from (i.e., [P] = person, [E] = environment, [O] = occupation)

Supplementary Table 3: Items in resulting 54-item TSOPP in order of difficulty.

	Item	Item Label	Item Difficulty Logit Measure	Standard Error	MnSq	Zstd
Items which are less difficult to manage	Managing/avoiding other substances	MANSUBST	1.85	0.07	1.23	2.22
	Getting to the exam hall	GETEXAMH	1.39	0.06	1.06	0.75
	Using computers	USECOMP	1.33	0.06	1.13	1.72
	Managing alcohol intake	MANALCOH	1.18	0.06	1.21	2.85
	Managing housemates	MANHOUSE	1.04	0.06	1.21	2.83
	Managing medication	MEDICATI	1.04	0.05	1.11	1.68
	Understanding the Library system	LIBSYSTE	0.84	0.05	1.17	2.70
	Managing Tutor System/Student Adviser system	MANTUTOR	0.81	0.05	1.04	0.68
	Managing lab/placement environments	MANLABPL	0.79	0.05	1.06	1.01
	Managing shopping, housework etc.	SHOPHOUSE	0.69	0.05	1.04	0.74
	Staying and doing the exam	STAYDOEX	0.67	0.05	1.18	3.04
	Doing practical work on placement (i.e., on placement, in labs)	PRACTICA	0.59	0.06	1.14	2.12
	Communicating with supervisor	COMMSUPE	0.46	0.05	0.92	-1.52
	Understanding the course structure and content	COURSTRU	0.44	0.05	0.94	-1.17
	Deciding which question to do	DECQUES	0.38	0.05	0.80	-4.14
	Managing anger	MANANGER	0.34	0.05	0.95	-0.95
	Managing finances	MANFINAN	0.32	0.05	1.04	0.87
	Being on time for college (lectures, labs etc.)	ONTIMECO	0.28	0.05	1.15	3.06
	Managing family	MANFAMIL	0.27	0.05	1.03	0.71
	Understanding topic/question	TOPIC	0.27	0.05	0.84	-3.37
	Understanding the content of lectures	UNDERCON	0.21	0.05	0.75	-5.52
	Communicating with people	COMMPEOP	0.17	0.05	0.96	-0.80
	Referencing	REFERENC	0.16	0.05	1.03	0.54
	Managing nutritional needs	NUTRITNE	0.11	0.05	1.07	1.38
	Working in groups	WORKGROU	0.10	0.05	0.93	-1.41
	Getting involved in societies	INVOLVES	0.04	0.05	1.23	4.44
	Understanding your departments expectations/standards (e.g., length, style etc.)	DEPARTEX	0.02	0.05	0.90	-2.17
	Taking notes in class	TAKENOTE	0.02	0.05	1.04	0.78
	Participating in discussion	PARTDISC	-0.06	0.05	1.14	2.69
	Tolerating external distractions e.g., noise, light	TOLERATE	-0.12	0.05	1.14	2.81
	Asking for help	ASKHELP	-0.15	0.05	0.97	-0.57
	Doing presentations	PRESENTA	-0.28	0.05	1.22	4.10
	Switching off and relaxing	SWITCHOF	-0.29	0.05	1.25	4.73
	Writing study notes after class	NOTEASFT	-0.30	0.05	1.04	0.78
	Managing panic and writer's block	MANPANIC	-0.31	0.05	0.97	-0.55
	Remembering what I have studied	REMSTUDY	-0.32	0.05	0.88	-1.43
	Organising information	ORGANISE	-0.33	0.05	0.98	-0.49
	Receiving and coping with bad results	RECBADRE	-0.33	0.05	0.92	-1.68

Items which are more difficult to manage	Concentrating during lectures and tutorials	CONCENLE	-0.39	0.05	0.82	-3.96
	Structuring and planning the essay or project	STRUCTPL	-0.49	0.05	0.87	-2.63
	Being a perfectionist	PERFECTI	-0.57	0.05	1.21	3.96
	Managing stress before an exam	MANSTREE	-0.62	0.05	1.07	1.35
	Knowing best how to study	KNOWBEST	-0.64	0.05	0.82	-3.65
	Managing my free time	MANFREET	-0.66	0.05	1.00	-0.03
	Getting enough good quality sleep	QUALSLEE	-0.72	0.05	1.19	3.48
	Being confident	BECONFID	-0.76	0.05	0.94	-1.12
	Getting down to writing	GETDOWNW	-0.83	0.05	0.97	-0.60
	Achieving goals	ACHIEVEG	-0.88	0.05	0.84	-3.05
	Maintaining mental stamina/ endurance	MENSTAMI	-0.94	0.05	0.82	-3.39
	Dealing with time pressures and deadlines	PRESSDEA	-1.02	0.05	0.91	-1.70
	Managing anxiety	MANANXIE	-1.09	0.05	0.95	-0.92
	Maintaining concentration during study	CONCENST	-1.11	0.05	0.77	-4.47
	Procrastination	PROCRAST	-1.28	0.05	1.17	2.75
	Dealing with work overload	WORKOVER	-1.31	0.05	0.75	-4.57

Key: *MnSq* = mean square fit statistics; *Zstd* = standardised mean square fit statistics
